# Supplementary material for: Sacubitril/Valsartan in Heart Failure with Reduced Ejection Fraction: Real-World Experience from Italy (the REAL.IT Study)
Source: J Clin Med. 2023 Jan 16;12(2):699. doi: 10.3390/jcm12020699 (PMC9863394; doi:10.3390/jcm12020699)
Supplement: Supplementary file 1 [file jcm-12-00699-s001.zip › Supplementary Table S1.pdf]

Table S1 Secondary and Exploratory Objectives of the REAL.IT Study.

**Secondary Objectives**
Evolving baseline pharmacotherapy and clinical characteristics in patients that initiate sacubitril/valsartan treatment, overall and by calendar quarter (relating to time since launch)
Evaluate resource utilization in patients that initiate sacubitril/valsartan treatment, overall and by calendar quarter (relating to time since launch) *
Describe sacubitril/valsartan drug utilization, including daily dose, titration dose, add-on, and persistence
Proportion and reason for not achieving target titration dose, overall and by calendar quarter (relating to time since launch)
Proportion, timing, and reason for sacubitril/valsartan discontinuation
Evaluate the frequency of cardiovascular (CV) and non-CV death, overall and by calendar quarter (relating to time since launch)
**Explorative objectives**
Evaluate adherence to treatment
Evaluate changes in functional capacity, systolic function, and the effect of treatment with sacubitril/valsartan on cardiac function indices, i.e., on systolic and diastolic function and on left ventricular remodeling indices (where available), overall and by calendar quarter (relating to time since launch)
Evaluate change in uric acid levels overall and by calendar quarter (relating to time since launch)

*Sodium-glucose co-transporter 2 (SGLT2) inhibitors (SGLT2i), now included as a component of the European Society of Cardiology (ESC) guidelines for the treatment of HFrEF, were not approved for HF treatment in Italy at the time of this study.
