# Supplementary material for: Sacubitril/Valsartan in Heart Failure with Reduced Ejection Fraction: Real-World Experience from Italy (the REAL.IT Study)
Source: J Clin Med. 2023 Jan 16;12(2):699. doi: 10.3390/jcm12020699 (PMC9863394; doi:10.3390/jcm12020699)
Supplement: Supplementary file 1 [file jcm-12-00699-s001.zip › Supplementary Table S2.pdf]

Table S2 Demographic and clinical characteristics at baseline for patients stratified by calendar quarter of inclusion.

**Year**	**2017**			**2018**				**2019**	
**Calendar Period**	**2**	**3**	**4**	**1**	**2**	**3**	**4**	**1**	**2**
Patients by calendar quarter, n	52	95	171	166	132	61	85	57	48
Age, (years) mean ± SD	64.6 (10.3)	63.4 (11.4)	63.6 (11.5)	63.5 (11.9)	63.7 (11.0)	64.2 (12.7)	66.7 (12.4)	65.7 (12.8)	69.1 (12.2)
Male, n (%)	44 (84.6)	79 (83.2)	143 (83.6)	145 (87.3)	113 (85.6)	56 (91.8)	68 (80.0)	47 (82.5)	40 (83.3)
Patients with available data on HF disease, n (%)	46 (88.5)	57 (60.0)	110 (64.3)	87 (52.4)	80 (60.6)	37 (60.7)	58 (68.2)	33 (57.9)	20 (41.7)
Duration of HF disease (years), mean ± SD	7.6 (7.5)	8.0 (6.1)	6.6 (6.1)	6.6 (5.7)	6.5 (6.1)	5.6 (5.0)	7.5 (5.1)	6.4 (6.8)	6.1 (6.5)
Patients with available data on clinical characteristics, n (%)	40 (76.9)	39 (41.1)	75 (43.9)	65 (39.2)	63 (47.7)	39 (63.9)	70 (82.4)	53 (93.0)	47 (97.9)
Ischemic heart disease, n (%)	13 (32.5)	19 (48.7)	34 (45.3)	21 (32.3)	24 (38.1)	14 (35.9)	22 (31.4)	9 (17.0)	15 (31.9)
PCI/CABG, n (%)	10 (25.0)	17 (43.6)	29 (38.7)	17 (26.2)	22 (34.9)	8 (20.5)	16 (22.9)	7 (13.2)	10 (21.3)
Diabetes mellitus, n (%)	9 (22.5)	6 (15.4)	29 (38.7)	15 (23.1)	14 (22.2)	9 (23.1)	13 (18.6)	NI	6 (12.8)
Hypertension, n (%)	16 (40.0)	16 (41.0)	45 (60.0)	29 (44.6)	29 (46.0)	22 (56.4)	29 (41.4)	13 (24.5)	9 (19.1)
Patients with available data on clinical examination, n (%)	48 (92.3)	90 (94.7)	165 (96.5)	147 (88.6)	118 (88.5)	49 (89.4)	56 (65.9)	30 (52.6)	15 (31.3)
NYHA class II, n (%)	28 (58.3)	35 (38.9)	125 (75.8)	94 (63.9)	85 (72.0)	29 (59.2)	38 (67.9)	18 (60.0)	10 (66.7)
NYHA class III, n (%)	18 (37.5)	54 (60.0)	39 (23.6)	53 (36.7)	33 (28.0)	18 (36.7)	18 (32.1)	10 (33.3)	5 (33.3)

Abbreviations: CABG = coronary artery bypass grafting; CRT = cardiac resynchronization therapy; HF = heart failure; ICD = implantable cardioverter defibrillator; NYHA = New York Heart Association; PCI = percutaneous coronary intervention; SD = standard deviation.
